# Rate of Decline in Serum PFOA Concentrations after Granular Activated Carbon Filtration at Two Public Water Systems in Ohio and West Virginia

**DOI:** 10.1289/ehp.0901252

**Published:** 2009-10-22

**Authors:** Scott M. Bartell, Antonia M. Calafat, Christopher Lyu, Kayoko Kato, P. Barry Ryan, Kyle Steenland

**Affiliations:** 1 Program in Public Health, University of California, Irvine, California, USA; 2 Department of Environmental and Occupational Health, Emory University, Atlanta, Georgia, USA; 3 Centers for Disease Control and Prevention, Atlanta, Georgia, USA; 4 Battelle/Centers for Public Health Research and Evaluation, Durham, North Carolina, USA

**Keywords:** exposure, half-life, intervention, perfluorooctanoic acid

## Abstract

**Background:**

Drinking water in multiple water districts in the Mid-Ohio Valley has been contaminated with perfluorooctanoic acid (PFOA), which was released by a nearby DuPont chemical plant. Two highly contaminated water districts began granular activated carbon filtration in 2007.

**Objectives:**

To determine the rate of decline in serum PFOA, and its corresponding half-life, during the first year after filtration.

**Methods:**

Up to six blood samples were collected from each of 200 participants from May 2007 until August 2008. The primary source of drinking water varied over time for some participants; our analyses were grouped according to water source at baseline in May–June 2007.

**Results:**

For Lubeck Public Service District customers, the average decrease in serum PFOA concentrations between May–June 2007 and May–August 2008 was 32 ng/mL (26%) for those primarily consuming public water at home (*n* = 130), and 16 ng/mL (28%) for those primarily consuming bottled water at home (*n* = 17). For Little Hocking Water Association customers, the average decrease in serum PFOA concentrations between November–December 2007 and May–June 2008 was 39 ng/mL (11%) for consumers of public water (*n* = 39) and 28 ng/mL (20%) for consumers of bottled water (*n* = 11). The covariate-adjusted average rate of decrease in serum PFOA concentration after water filtration was 26% per year (95% confidence interval, 25–28% per year).

**Conclusions:**

The observed data are consistent with first-order elimination and a median serum PFOA half-life of 2.3 years. Ongoing follow-up will lead to improved half-life estimation.

The Washington Works plant in Washington, West Virginia, is owned and operated by the DuPont Company. Since 1951, the facility has used perfluorooctanoate (PFOA) and related materials. PFOA contamination of water supplies downwind and downstream from the Washington Works plant has been observed since the 1980s and is thought to be attributable to historical releases from the facility. Despite a 99% reduction in air and water PFOA emissions from the facility between 2000 and 2006, groundwater supplies near the facility remain contaminated with PFOA. In 2007, two nearby public water districts began routine treatment with granular activated carbon to remove PFOA from the potable water supply. Lubeck Public Service District in West Virginia began filtration on 4 June 2007. Little Hocking Water Association in Ohio began filtration on 2 November 2007. These interventions were designed to reduce the levels of PFOA in drinking water, which had previously been shown to be a major source of exposure in the community (Emmett et al. 2006). With a dual filter design, careful monitoring for breakthrough, and frequent filter changes, activated carbon filtration is highly effective at removing PFOA. During our study period, prefiltration water PFOA concentrations for Little Hocking ranged from 1.9 to 4.9 ng/mL (parts per billion) and postfiltration water PFOA concentrations were all nonquantifiable (< 0.016 ng/mL) or nondetectable (Little Hocking Water Association 2008). For Lubeck, PFOA concentrations in drinking water samples between 2001 and 2006 ranged from 0.41 to 1.0 ng/mL (Peer Consultation Panel 2008). Postfiltration water PFOA concentrations in Lubeck have also been reported to be less than the quantification limit.

Although PFOA was long thought to be biologically inactive, studies have shown hepatic toxicity, carcinogenicity, reproductive toxicity, hormone disruption, and immunotoxicity in controlled experiments using laboratory animals (Lau et al. 2007). Epidemiologic studies of exposed workers have generally failed to show clear mortality or cancer effects, but some have suggested lipid and hormone effects (Costa et al. 2009). Rat studies suggest that PFOA is easily absorbed through the gastrointestinal tract, binds to serum albumin, is metabolically inert, and is excreted primarily through the kidneys (Vanden Heuvel et al. 1991). Bolus dose experiments in laboratory animals suggest plasma half-lives ranging from 3 hr to about 1 month, varying by species and sex, with a remarkably short half-life in female rats (Vanden Heuvel et al. 1991). One unusual feature of PFOA is that serum rapidly reaches steady state with daily dosing but that elimination occurs more slowly. Researchers have recently proposed toxicokinetic models for PFOA with two or more compartments and time-dependent binding and elimination rates (Anderson et al. 2006; Tan et al. 2008). After exposure is ceased or is diminished, a one-compartment model reportedly fits the limited available human data reasonably well. In a 5-year study, Olsen et al. (2007) observed an average serum half-life of 3.8 years among 24 male and 2 female retired workers with previous occupational exposures and a baseline median serum PFOA concentration of 408 ng/mL. However, they did not examine age or sex dependence in elimination rates. Moreover, complex elimination patterns predicted by a multicompartment model are unlikely to be observed in a cohort without recent exposures.

Declines of 10–20% per year from baseline serum PFOA concentrations of about 22–25 ng/mL were recently reported for a German cohort of 164 mothers, 90 children, and 100 men after residential drinking water filtration began in 2006 (Hölzer et al. 2009). The first round of blood samples was collected several months after filtration, and a second sample was collected from each participant 335–386 days after the first sample. Although these rates of decrease correspond to half-lives of approximately 3–7 years, their interpretation is complicated by relatively low baseline serum PFOA concentrations and several periods of filter breakthrough during which PFOA exposures resumed. Both factors are expected to cause serum PFOA removal rates to be underestimated and half-lives to be overestimated.

Study of pre- and postfiltration serum PFOA concentrations among residents living near the Washington Works facility has several advantages for understanding PFOA toxicokinetics in humans. Female and male participants with diverse ages can easily be included, baseline serum concentrations are far above U.S. background levels, and potential nonlinear elimination patterns can be assessed by intensive postfiltration sampling.

In the present report, we describe our first year of follow-up of individuals from the Lubeck Public Service District and Little Hocking Water Association. Our analyses are based on interviews and blood samples before and after filtration. We also describe the effectiveness of water filtration and findings regarding PFOA toxicokinetics during the first year.

## Materials and Methods

In 2007, 200 adults were recruited from the C8 Health Project to participate in an ongoing study of PFOA toxicokinetics. The C8 Health Project, conducted in 2005–2006, was a cross-sectional study of approximately 69,000 individuals who had lived in at least one of six affected water districts near the Washington Works plant; participants provided a blood sample and answered a questionnaire regarding residential and occupational history, water use, health history, and demographic information (Frisbee et al. 2009; Steenland et al. 2009). The C8 Health Project was initiated as a result of the settlement agreement in a class action lawsuit filed in West Virginia (Leach *v*. E.I. du Pont de Nemours & Co. 2002). The settlement also appointed a group of epidemiologists, the C8 Science Panel, to assess whether a link between PFOA exposure and disease in the community exists (C8 Science Panel 2009).

Using phone numbers reported during the C8 Health Project, we attempted to contact eligible individuals for our study of PFOA toxicokinetics by telephone in May 2007 to ask additional eligibility screening questions. The random sample was stratified by sex, water service provider, and primary drinking water source (bottled vs. public). By design, approximately 75% of our participants were recruited from Lubeck to allocate most study resources to observing short-term elimination patterns because filtration began there soon after our baseline interviews. The remaining 25% were recruited from Little Hocking, which did not begin filtration until about 5 months after our baseline interviews. These participants served as a comparison group to assess short-term serum PFOA patterns in the absence of filtration. We recruited approximately equal numbers of males and females to maximize statistical power for detecting any sex differences. Although most of our participants were selected on the basis of consuming public drinking water, our recruitment targets included a small number of bottled water drinkers (20 participants in Lubeck and 10 in Little Hocking) to investigate the rate of decline in their PFOA serum concentrations. Up to six attempts were made to contact each randomly selected individual, including calls on weekdays and weekends and during daytime and early evenings, until the individual was reached or refused to speak with us, or until the recruitment goal had been reached for that individual’s stratum.

We attempted to contact 1,092 individuals before reaching our recruitment goals. Of these, 94 had no working phone number reported to the C8 Health Project, 435 had working phone numbers but could not be reached within six attempts, and 46 refused to answer our questions or were not able to complete the telephone interview. Of the 517 individuals who completed our screening interview, 304 were ineligible based on the additional eligibility screening questions, and 7 were unavailable during the first round of blood sampling or refused to participate in the full study. An additional 6 individuals were initially retained as backup participants but were not followed after our 200 primary study participants completed their first blood draw.

Based on 2005–2006 C8 Health Project data, we used the following eligibility criteria for the study of PFOA toxicokinetics: serum PFOA concentration of at least 50 ng/mL, residential water service provided by Lubeck Public Service District or Little Hocking Water Association, not growing their own vegetables at home, never employed by the DuPont Company, and a signed consent form allowing C8 Health Project data to be shared with the C8 Science Panel. Two groups were recruited: those reporting primary use of public water for water consumption at home, and a smaller group consisting of those primarily using bottled water for residential water consumption. To be eligible, participants had to have reported primary use of public water for water consumption at home in 2005–2006 and in 2007; primary use of public water for cooking, showering, and bathing in 2007; and no use of carbon water filters at home. Eligible bottled water drinkers had to have reported primary use of bottled water for residential water consumption both in 2005–2006 and in 2007. Those who were not served by the same water district in 2007 as in 2005–2006 or those with any history of DuPont employment or likely occupational PFOA exposures since 2000 were excluded from enrollment for both groups. A total of 172 eligible public water drinkers and 28 eligible bottled water drinkers consented to enroll in the study. The ages of the participant ranged from 18 to > 89 years. Table 1 lists baseline characteristics of enrolled participants.

Participants were asked to donate eight blood samples over 4 years, beginning in May 2007. Collection of the first round of blood samples was completed in June 2007. Subsequent blood draws took place approximately 1, 2, 3, 6, and 12 months after the initial donation, with the final sample donated in August 2008. A brief questionnaire regarding recent water use, local fruit/vegetable consumption, and potential occupational exposure was administered by computer-assisted telephone interview before each blood draw. All but 3 of the original 200 participants remained enrolled throughout the first year of the study, completing all six interviews and blood samples. Additional blood draws are planned for mid-2009 and mid-2011.

Blood samples (~ 4 mL) were collected at each visit by trained phlebotomists at a location of the participant’s choosing, generally at home. Samples were transported in coolers to a laboratory on the same day, allowed to clot at room temperature for a minimum of 30 min, and centrifuged for at least 15 min at 2,400 rpm. After centrifuging, serum was transferred to 2 mL polypropylene Nalgene vials (Thermo Fisher Scientific, Waltham, MA) and stored at −30°C for up to 1 week before being shipped on dry ice to the Centers for Disease Control and Prevention (CDC).

At the CDC, all samples were kept at or below −40°C until analysis. Concentrations of PFOA in serum were measured using a modification of online solid-phase extraction (SPE) coupled to reverse-phase high-performance liquid chromatography (HPLC)–isotope dilution tandem mass spectrometry previously described (Kuklenyik et al. 2005). Briefly, we added 275 μL 0.1 M formic acid and 25 μL ^13^C_2_^−^ PFOA internal standard solution to 100 μL serum. The spiked serum was vortex mixed and sonicated and then injected into a Symbiosis online SPE-HPLC system (Spark Holland, Plainsboro, NJ), allowing for the preconcentration of PFOA on a C18 SPE column. This column was automatically positioned in front of a Betasil C8 analytical HPLC column (2.1 × 50 mm, 5 μm; ThermoHypersil-Keystone, Bellefonte, PA) for the chromatographic identification of PFOA on a Surveyor HPLC system (ThermoFinnigan, San Jose, CA) operated at a 300 μL/min flow rate with 20 mM ammonium acetate (pH 4) in water (mobile phase A) and acetonitrile (mobile phase B). The HPLC gradient program (11 min) started at 10% of mobile phase B and increased from 10% to 55% (0.5–4.0 min), from 55% to 80% (4–7.0 min), and from 80% to 90% (7–9 min), then B content 90% hold (0.5 min), decreased to 10%, and hold 10% (9.6–11 min). Detection and quantification used negativeion heated electrospray ionization (a variant of electrospray ionization) tandem mass spectrometry on a ThermoFinnigan TSQ Quantum Ultra triple-quadrupole mass spectrometer. The isotope-labeled internal standard used for quantification was ^13^C_2_-PFOA. We used 10 PFOA calibration standards, spiked into calf serum, encompassing the entire linear range of the method (0.1–150 ng/mL), to construct a daily calibration curve for quantification. For samples with PFOA concentrations higher than the highest calibration standard, we used a volume of serum of < 100 μL, so the actual volume of serum used varied between 10 μL and 100 μL, depending on the sample. In addition to the calibration standards, we analyzed blanks and low-concentration (~ 4 ng/mL), medium-concentration (~ 17 ng/mL), and high-concentration (~ 100 ng/mL) quality control (QC) materials, prepared in calf serum, along with each batch of samples to ensure the accuracy and reliability of the data across time (Kuklenyik et al. 2005). The concentrations of the QC materials, averaged to obtain one measurement each of high-, medium-, and low-concentration QC for each batch, were evaluated using standard statistical probability rules (Caudill et al. 2008).

Duplicate 4 mL blood samples were collected from 32 participants during the first round of blood draws in May–June 2007. Spun serum from these samples was shipped to the Southwest Research Institute (San Antonio, TX) as a quality assurance check and analyzed using liquid chromatography–tandem mass spectrometry; the Spearman rank correlation coefficient for paired PFOA concentrations measured by the two laboratories was 0.89. The median ratio between paired concentrations was 1.16, with higher average estimates obtained by the CDC laboratory.

Several of the blood samples collected by a single phlebotomist during the month 2 visit appear to have implausible serum PFOA concentrations compared with PFOA measurements for the same individuals at other time points. We strongly suspect that these blood samples were inadvertently mislabeled during collection or processing before shipment to the CDC. Thus, all 19 blood samples collected by this phlebotomist during the month 2 visit were excluded from the analysis.

All statistical analyses were performed using R (R Development Core Team 2008). Unless otherwise noted, statistics were calculated using all available valid observations; that is, participants who dropped out of the study and those with omitted 2-month measurements were not excluded from the analyses. Baseline comparisons were conducted using unpaired two-sample *t*-tests using unequal variances for continuous variables, and chi-square tests with continuity corrections for proportions. All reported *p*-values reflect two-tailed hypothesis tests.

Linear mixed effects models were used to assess subject-specific changes in serum PFOA concentrations over time and to estimate serum PFOA half-lives. The following general model form was used to describe the observed data:


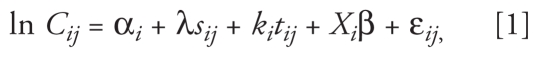


where *C_ij_* is the serum PFOA concentration measured for individual *i* at sampling round *j*, α*_i_* is the subject-specific intercept, λ is the prefiltration slope, *s_ij_* is the prefiltration duration (i.e., time elapsed between blood sample collection and the first day of filtration, set to 0 after filtration), *k_i_* is the subject-specific postfiltration slope, *t_ij_* is the postfiltration duration (i.e., time elapsed between the first day of filtration and the blood sample collection day, set to 0 before filtration), *X_i_* is a vector of fixed covariates for individual *i*, β is a vector of fixed effects corresponding to the covariates, and ɛ*_ij_* is a random error term. The intercept, postfiltration slope, and error terms were all modeled as random effects with normal distributions; other parameters were treated as fixed effects. The prefiltration slope might also be expected to vary across subjects but could not be estimated as a random effect due to a lack of multiple prefiltration blood samples for most of our subjects. This model assumes that log serum PFOA concentrations were changing linearly over time for a few weeks before filtration for the Lubeck measurements, and for several months before filtration for the Little Hocking measurements. It also assumes a linear postfiltration slope in log serum PFOA concentrations over time for each individual. Parameter estimation was accomplished using the lme package in R, with maximization of the restricted log-likelihood.

Our longitudinal analyses using the linear mixed effects models included the following covariates as predictors: water district, public/bottled water drinker, sex, age, local produce consumer, homegrown produce consumer, and public water service at work. These covariates were selected because average serum PFOA concentrations measured by the C8 Health Project were higher in those served by Little Hocking than for Lubeck, and because related factors (i.e., age, tap water drinks per day, servings of homegrown fruit and vegetables) have been reported to be predictors of serum PFOA concentrations among residents served by Little Hocking Water Association (Emmett et al. 2006). In addition, an interaction term was used between water district and water source (public/bottled water drinker), in order to allow the effect of water source on baseline PFOA to differ by water district. PFOA concentration was the only time-varying measurement entered into the models; baseline values were used for all other covariates. Model assumptions were evaluated using diagnostic plots and by testing the addition of quadratic terms for *t_ij_* and age.

Notably, our mixed effects model is equivalent to assuming first-order elimination kinetics with one compartment after filtration, if postfiltration PFOA exposure rates are negligible relative to baseline PFOA serum concentrations. The median PFOA serum concentration in the U.S. general population is 4 ng/mL (Calafat et al. 2007), a value about 15–100 times smaller than the mean baseline serum concentrations in our four study groups. By definition, after exposures cease first-order elimination is described using the following equation:


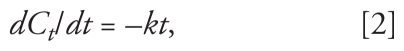


where *C_t_* is the concentration at time *t*, and *k* is the elimination rate constant. The solution to this equation is well known:


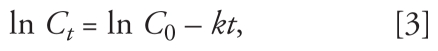


which has the same form as the linear mixed effects model used in this study, because *s_ij_* = 0 for all postfiltration measurements. If postfiltration exposures are negligible, α*_i_* + *X_i_*β represents the baseline log serum PFOA concentration for participant *i*, − *k_i_* represents the subject-specific postfiltration elimination rate constant, and the subject-specific serum half-life is −ln(2)/*k_i_*. Because the serum PFOA concentrations appear to be lognormally distributed, the log transformation is appropriate for both the mean model and for the residuals.

## Results

Table 1 shows baseline summary statistics. Baseline (May–June 2007) serum PFOA concentrations ranged from 16 to 1,400 ng/mL and were substantially higher for Little Hocking Water Association customers than for Lubeck Public Water District customers (*t*-test; *p* < 0.001). Baseline PFOA concentrations were higher for those reporting primary use of public water for drinking water at home compared with those primarily using bottled water, both for Lubeck (*p* < 0.001) and for Little Hocking (*p* = 0.004). Consuming homegrown produce was reported by more bottled water drinkers than by public water drinkers in Little Hocking (*p* = 0.035) but not in Lubeck (*p* = 0.46). Other baseline characteristics (sex, age, proportion provided public water at work, and proportion eating local produce) did not differ significantly between public and bottled water drinkers in either locality (Table 1).

**Figure 1:**
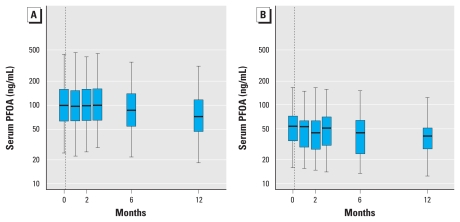
Box-and-whisker plots of serum PFOA concentrations at baseline and approximately 1, 2, 3, 6, and 12 months after baseline for participants with residential water service from Lubeck Public Service District, stratified by primary drinking water source at baseline (*A*, public water; *B*, bottled water). Boxes mark the 25th, 50th, and 75th percentiles of measured serum PFOA for each round of blood samples, and whiskers extend to the lowest and highest measured concentrations. The vertical dashed line indicates the day on which water filtration began, relative to the median day of baseline blood sample collection. Serum PFOA concentrations are plotted on a log scale.

**Figure 2:**
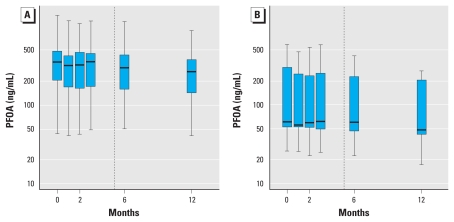
Box-and-whisker plots of serum PFOA concentrations at baseline and approximately 1, 2, 3, 6, and 12 months after baseline for participants with residential water service from Little Hocking Water Association, stratified by primary drinking water source at baseline (*A*, public water; *B*, bottled water). Boxes mark the 25th, 50th, and 75th percentile of measured serum PFOA for each round of blood samples, and whiskers extend to the lowest and highest measured concentrations. The vertical dashed line indicates the day on which water filtration began, relative to the median day of baseline blood sample collection. Serum PFOA concentrations are plotted on a log scale.

During the first year, serum PFOA concentrations declined for residential customers of both water systems (Figures 1 and 2). For Lubeck residential customers, the mean unadjusted decrease in serum PFOA concentrations between May–June 2007 and May–August 2008 was 32 ng/mL [95% confidence interval (CI), 27–37 ng/mL] for those primarily consuming public water at home (*n* = 130), and 16 ng/mL (95% CI, 10–22 ng/mL) for those primarily consuming bottled water at home (*n* = 17). For Little Hocking customers, the mean unadjusted decrease in serum PFOA concentrations between May–June 2007 and May–June 2008 was 95 ng/mL (95% CI, 62–129 ng/mL) for public water consumers (*n* = 39) and 68 ng/mL (95% CI, 1.5–135 ng/mL) for bottled water consumers (*n* = 11). These decreases comprise 26%, 28%, 22%, and 38%, respectively, compared with the mean baseline serum PFOA concentrations for the four groups. For blood samples collected after filtration began in Little Hocking, from November–December 2007 to May–June 2008, the average unadjusted decrease in serum PFOA was 39 ng/mL (11% change from the November–December mean) for public water consumers and 28 ng/mL (20% change from the November–December mean) for bottled water consumers. We also calculated unadjusted individual percentage changes in serum PFOA (summarized in Table 2). The means of the individual percentage declines differ slightly from the relative changes in the group means, because the two calculations are not equivalent.

Table 3 shows results from the linear mixed effects model and displays the exponentiated parameter estimates. For example, elapsed postfiltration time was associated with a decrease of 0.31 units in log serum PFOA per year after adjusting for other covariates (*e*^0.31^ = 0.74), indicating that the average effect of time since filtration was to decrease the serum PFOA concentration to 74% of its previous value (a 26% decrease) each year. This elimination rate estimate is equivalent to an average half-life of 2.3 years (95% CI, 2.1–2.4 years). Age at baseline also had a strong effect on serum PFOA concentrations, with an average increase of 16% per 10 years of age. Sex, consumption of homegrown or locally grown vegetables, and a public water supply at work were not significantly associated with log serum PFOA concentrations. Water district (*p* = 0.02) and public/bottled drinking water source (*p* < 0.001) were significantly associated with log PFOA after adjusting for the other covariates. The interaction term between water district and public/drinking water source had a large magnitude but was not statistically significant (*p* = 0.10). We estimated the between-individual SD in subject-specific postfiltration elimination rate constants to be 0.080 units/year using our mixed effects model.

We also used the mixed effects model described in Table 3 to test the hypothesis that serum PFOA declined more quickly after filtration began. Because serum PFOA concentrations appear to have already been declining before filtration, we used the difference between prefiltration and postfiltration elimination rates as a test statistic. The estimated difference in log PFOA elimination rates was 0.11 units/year (95% CI, 0.036–0.19 units/year). This contrast was statistically significant (*p* = 0.004), supporting granular activated carbon filtration as an effective intervention in this setting. In relative terms, filtration appears to have increased the rate of decline by about 60% during the first year.

We conducted additional analyses to determine whether elimination rates depended on certain covariates. These analyses were motivated by specific hypothesis but were somewhat exploratory in that they involved empirical model selection and were expected to have little statistical power because of the limited follow-up time and the general difficulty in detecting statistical interactions. Starting with the linear effects model described in Table 3, we added interaction terms for each of the fixed predictors with time since filtration, all at once. At this stage the only statistically significant effect modifiers for elimination rates were water district/water supply interaction (*p* = 0.03) and homegrown vegetable consumption (*p* < 0.001). The estimated effects were equivalent to a 28% higher average half-life (*p* = 0.13) in Lubeck versus Little Hocking, a 33% higher average half-life (*p* = 0.07) for public versus bottled water drinkers in Little Hocking, a 7.8% lower average half-life (*p* = 0.24) for public versus bottled water drinkers in Lubeck, and a 46% higher half-life among homegrown vegetable consumers (*p* < 0.001). Although the individual observed differences in serum PFOA removal rates between localities and water supply were not statistically significant and may simply reflect random variation, the significant effect of homegrown vegetable consumption on the apparent half-life is intriguing. The higher estimated half-life for homegrown vegetable consumers may indicate an ongoing PFOA exposure that is artificially inflating the half-life estimates for those individuals. One hundred two participants never reported homegrown vegetable consumption during any of the interviews in the first year. When we restricted the analysis to these individuals, the estimated median half-life (adjusting for the same variables as shown in Table 3 except for homegrown vegetable consumption) is 2.1 years (95% CI, 1.9–2.4 years). We found no evidence of age- or sex-dependence of the postfiltration elimination rates during the first year of the study.

We also evaluated the potential dependence of elimination rates on serum PFOA concentration itself by adding an interaction term between time after filtration and an indicator variable for baseline serum PFOA concentration above the median value to the linear effects model in Table 3; the average half-life estimates produced by this model were 14% higher (*p* = 0.07) for those with baseline serum PFOA concentrations above the median.

A quadratic term for age was of a small magnitude and not statistically significant (*p* = 0.52). This result suggests that the linearity assumption was reasonable for age as a continuous covariate.

## Discussion

Our analysis was consistent with previous findings associating serum PFOA concentrations with age, water district, and public/bottled water source in these communities (Emmett et al. 2006; Steenland et al. 2009). Although we did not replicate previously reported associations with homegrown produce consumption, we designed our study to exclude individuals who had previously reported growing their own produce. Thus, we may have omitted those with the highest homegrown produce consumption rates. Given this exclusion, we were surprised by the large number of individuals reporting homegrown produce consumption during the baseline interview in our study. This may have resulted from phrasing differences between our questionnaire (“Do you eat any fruits or vegetables grown at your own home?”) and the C8 Health Project instrument (“Do you grow your own vegetables?”). Many of our respondents may have been reporting consumption of fruits/vegetables grown by other family members or neighbors.

One interesting feature of our data is a similar, or perhaps even faster, rate of decline in log serum PFOA concentrations among bottled water drinkers than among public water drinkers during the first year of our study. Because the rate of decline is modeled on a log scale, equivalent parameters indicate that declines in serum PFOA are proportionate to baseline serum PFOA concentrations, with larger absolute declines in the public water groups that started with higher PFOA concentrations. The substantial declines among both bottled water and public water drinkers indicate that PFOA exposures have decreased in the recent past for both groups, whether due to filtration at the water systems, reduction in some other environmental exposure, or both. Indeed, the facts that both the prefiltration and postfiltration slopes for log PFOA versus time are negative and that the postfiltration slope is significantly steeper suggest that exposures were reduced among all groups in our study population both before our study and again after filtration. Previous exposure reductions may have occurred in our participants because of sharply reduced air and surface water emissions from the Washington Works plant over the last decade. Moreover, it seems likely that even those individuals reporting primary use of bottled water for drinking at home were likely to have been exposed to PFOA-contaminated water through other means such as drinking water at restaurants or churches or while visiting others’ homes, or use of public water for cooking or beverage preparation at home, so the filtration was an effective intervention even in the bottled water group. During our baseline interviews, 36% of our bottled water drinkers reported using public water for cooking and 54% reported using public water for brewing coffee/tea or for home mixed beverages. Some individuals also reported changing their primary drinking water source over time. For example, despite baseline characterizations, 39% of our “bottled water drinkers” reported using public water as the primary drinking water source at home by the 12-month interview, and 10% of our “public water drinkers” reported drinking primarily bottled water at the 6-month interview.

PFOA contamination of drinking water has only recently been recognized as a potential problem in these communities, so many bottled water drinkers in our study might have switched from public water recently, perhaps as a result of Little Hocking’s offer of free bottled water for its customers starting in 2005 (Hunt 2006). To be included in our bottled water group, participants had to report bottled water consumption both in the 2005–2006 C8 Health Project and in our 2007 baseline interview, indicating some history of bottled water consumption that likely led to lower serum PFOA concentrations by mid-2007 when our baseline blood samples were collected. Although we attempted to build more elaborate statistical models to investigate variability in prefiltration serum PFOA concentrations for bottled and public water systems, we had only a limited number of observations in Little Hocking and were unable to fit more complex prefiltration models. The observed patterns are consistent with the explanation of exposure reduction in multiple sources, including filtration at the two water systems. However, apparent patterns among the Little Hocking bottled water drinkers should be interpreted with caution, because they may be unstable given the very small sample size (*n* = 11).

Animal studies indicate substantial distribution of PFOA to the liver and kidneys (Vanden Heuvel et al. 1991), which suggests that two or more compartments may be necessary to describe the toxicokinetics of PFOA under fluctuating exposure conditions. Researchers have resorted to elaborate model structures (e.g., saturable resorption from filtrate) in order to explain short-term PFOA toxicokinetics in animals given relatively high doses (Lou et al. 2009; Tan et al. 2008), but these multicompartment models may not be necessary to predict PFOA toxicokinetics in humans with relatively low environmental exposures during the first year after exposure ceases. The results from the first year of our study were reasonably explained using a log-linear model, which implies first-order elimination after filtration. One possible departure from the simpler model that we observed in our data was a slightly concave function formed by adding a statistically significant (*p* < 0.001) quadratic term for *t_ij_*, which predicted a slower PFOA elimination in the first months after filtration, increasing by 12 months to the same elimination rate estimated by the linear model. This behavior is likely explained by the fact that water systems remained contaminated with PFOA to some extent for days to weeks after filtration began, due to contaminated water already being present in storage tanks and in the distribution systems. Managers at the Little Hocking Water Association and Lubeck Public Water District systems have speculated that it may have taken weeks or months for the systems to become free of PFOA, during which time our participants may have continued to be exposed via drinking water, albeit at ever decreasing rates.

One advantage of using the mixed effects model to estimate PFOA elimination rates in this setting is its robustness with respect to fixed effects specification. Because random subject- specific intercepts and slopes are included, the validity of the model does not depend on correctly identifying every fixed predictor of these characteristics. We allowed each individual’s baseline PFOA concentration and postfiltration elimination rate to vary according to the observed data. Another advantage of the mixed effects model formulation is the inherent shrinkage of subject-specific elimination rates toward the mean, providing more reliable estimation for subjects with extreme half-lives (Pinheiro and Bates 2000).

Olsen et al. (2007) estimated that the geometric mean serum elimination half-life of PFOA was 3.8 (95% CI, 3.1–4.4) years for a group of 26 retired workers with previous occupational exposures. In contrast, the mean log PFOA elimination rate estimated by our initial mixed effects model is equivalent to a half-life of 2.3 years (95% CI, 2.1–2.4 years). Although we found a relatively narrow CI for the mean half-life, it is heavily influenced by the 12-month serum PFOA measurements and should be therefore viewed as a preliminary estimate that will be improved by collection of later blood samples. Although our log-linear model appears to fit the available data well, our half-life estimate depends on the additional assumption that ongoing PFOA exposures only contribute negligible amounts to current serum PFOA concentrations. Olsen et al. (2007) used the same assumption to derive their half-life estimate. Nonnegligible postfiltration exposures may have occurred among some of our participants because of homegrown/local produce consumption, PFOA contaminated water consumption at work or other locations, or other exposure pathways. Although airborne exposure routes may have once contributed to PFOA contamination in this community, emissions from the Washington Works facility are reported to be negligible for the past few years, and most public water supplies in the area have been tested for PFOA and are now being treated if needed. It is therefore likely that ongoing exposures in this community are minimal at present, with the possible exception of local/homegrown produce consumption from contaminated soils.

In addition, the relatively large random effect for this parameter suggests that subject-specific half-lives vary widely. For example, based on our results, about 95% of individuals would be expected to have elimination rate constants between 0.15 and 0.46 units per year, corresponding to half-lives between 1.5 and 4.6 years. In comparison, Olsen et al. (2007) reported subject-specific PFOA half-lives ranging from 1.5 to 9.1 years among 26 subjects, without shrinkage estimation. If any of the individuals in either study were experiencing substantial PFOA exposures during follow-up, the half-lives for those subjects would be overestimated.

A wide variation in subject-specific half-lives has important implications for risk assessment, because it implies that some individuals will accumulate higher body burdens, and potentially greater health risks, than others at the same rate of exposure. It also has implications for epidemiologic studies that rely on measured serum concentrations to characterize exposure. Such studies will be hampered by measurement error unless factors that are predictive of subject-specific half-lives can be identified. Although our preliminary data offer only a limited ability to investigate the determinants of subject-specific half-lives, the combination of repeated questionnaires and blood samples used in our study design are expected to provide more useful data for investigating these issues over the next few years.

## Figures and Tables

**Table 1:** Baseline characteristics of enrolled participants in May–June 2007, by water district and primary source of drinking water (public water source or bottled water).

	Lubeck		Little Hocking		
Characteristic	Public	Bottled	Public	Bottled	All participants
No. enrolled	132	17	40	11	200
Percent female	49	59	50	55	50
Age (years)^a^	55.8 ± 15	52 ± 16	51.8 ± 14	53 ± 15	54.5 ± 15
Serum PFOA (ng/mL)^a^	122 ± 81	58 ± 36	424 ± 333	180 ± 193	180 ± 209
Percent provided with public water at work	33	29	45	9	34
Percent eating homegrown produce	30	18	18	55	28
Percent eating local produce	77	71	72	91	76

a Data are mean ± SD.

**Table 2:** Percentage change in individual serum PFOA concentrations (ng/mL) for enrolled participants, by baseline water district and water source.

	Lubeck		Little Hocking		
Statistic	Public	Bottled	Public	Bottled	All participants
Twelve months (summer 2007 to summer 2008)					
Mean	−26	−27	−23	−27	−26
SD	11	10	13	15	11
Minimum	−51	−47	−51	−55	−55
Maximum	13	−8	5	7	13
Last 6 months (November/December 2007 to summer 2008)					
Mean	−15	−14	−12	−15	−15
SD	10	17	9	13	11
Minimum	−38	−31	−34	−36	−38
Maximum	12	43	2	12	43

**Table 3:** Estimated multiplicative effects on serum PFOA concentration.

Covariate	Effect multiplier^a^	95% CI
Time before filtration (per year)	0.83	0.77–0.89
Time after filtration (per year)	0.74	0.72–0.75
Lubeck versus Little Hocking, bottled water	0.54	0.32–0.91
Lubeck versus Little Hocking, public water	0.33	0.26–0.42
Public versus bottled water, Little Hocking	3.20	1.99–5.03
Public versus bottled water, Lubeck	1.95	1.39–2.73
Male	1.05	0.86–1.27
Age per 10 years	1.16	1.09–1.25
Consumption of local vegetables	0.97	0.77–1.22
Consumption of homegrown vegetables	1.09	0.88–1.35
Public water supply at work	1.06	0.85–1.32

a Effects are calculated from exponentiated coefficients in a log PFOA linear mixed effects model adjusting for water district and water source in combination and all of the other covariates listed in the table. Baseline responses were used for all covariates.
